# PV-specific loss of the transcriptional coactivator PGC-1α slows down the evolution of epileptic activity in an acute ictogenic model

**DOI:** 10.1152/jn.00295.2021

**Published:** 2021-11-17

**Authors:** Connie Mackenzie-Gray Scott, R. Ryley Parrish, Darren Walsh, Claudia Racca, Rita M. Cowell, Andrew J. Trevelyan

**Affiliations:** ^1^Medical School, Newcastle University Biosciences Institute, Newcastle upon Tyne, United Kingdom; ^2^Department of Neuroscience, Drug Discovery Division at Southern Research, Birmingham, Alabama; ^3^Department of Cell, Developmental and Integrative Biology, University of Alabama at Birmingham, Birmingham, Alabama

**Keywords:** neocortex, parvalbumin, PGC-1α, preictal, seizure

## Abstract

The transcriptional coactivator, PGC-1α (peroxisome proliferator-activated receptor γ coactivator 1α), plays a key role in coordinating energy requirement within cells. Its importance is reflected in the growing number of psychiatric and neurological conditions that have been associated with reduced PGC-1α levels. In cortical networks, PGC-1α is required for the induction of parvalbumin (PV) expression in interneurons, and PGC-1α deficiency affects synchronous GABAergic release. It is unknown, however, how this affects cortical excitability. We show here that knocking down PGC-1α specifically in the PV-expressing cells (PGC-1α^PV−/−^) blocks the activity-dependent regulation of the synaptic proteins, SYT2 and CPLX1. More surprisingly, this cell class-specific knockout of PGC-1α appears to have a novel antiepileptic effect, as assayed in brain slices bathed in 0 Mg^2+^ media. The rate of occurrence of preictal discharges developed approximately equivalently in wild-type and PGC-1α^PV−/−^ brain slices, but the intensity of these discharges was lower in PGC-1α^PV−/−^ slices, as evident from the reduced power in the γ range and reduced firing rates in both PV interneurons and pyramidal cells during these discharges. Reflecting this reduced intensity in the preictal discharges, the PGC-1α^PV−/−^ brain slices experienced many more discharges before transitioning into a seizure-like event. Consequently, there was a large increase in the latency to the first seizure-like event in brain slices lacking PGC-1α in PV interneurons. We conclude that knocking down PGC-1α limits the range of PV interneuron firing and this slows the pathophysiological escalation during ictogenesis.

**NEW & NOTEWORTHY** Parvalbumin expressing interneurons are considered to play an important role in regulating cortical activity. We were surprised, therefore, to find that knocking down the transcriptional coactivator, PGC-1α, specifically in this class of interneurons appears to slow ictogenesis. This anti-ictogenic effect is associated with reduced activity in preictal discharges, but with a far longer period of these discharges before the first seizure-like events finally start. Thus, PGC-1α knockdown may promote schizophrenia while reducing epileptic tendencies.

## Graphical abstract

**Figure:**
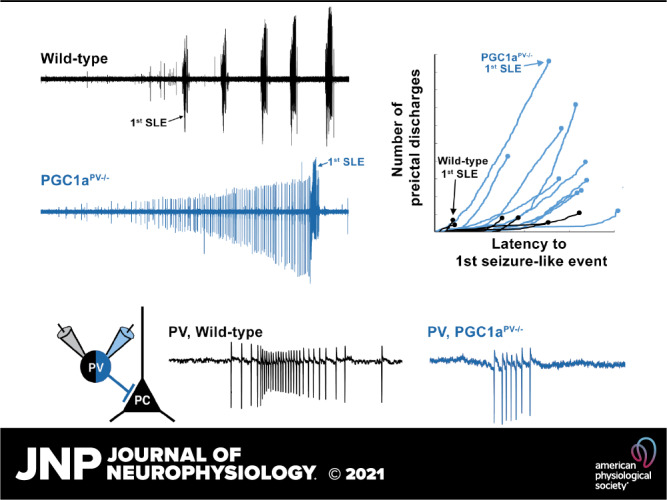

## INTRODUCTION

Studies in nonneuronal tissue have identified the transcriptional coactivator, PGC-1α (peroxisome proliferator-activated receptor γ coactivator 1α), as a key regulator of expression of nuclear-encoded mitochondrial genes (1–4). Its expression is induced by various triggers, including exercise, cold, and fasting, and it then drives mitochondrial (5) and peroxisome biogenesis (6) and uncouples mitochondrial respiration (7). PGC-1α is also highly expressed in different parts of the brain, where its expression is enriched in GABAergic neurons (8). Within cortical networks, PGC-1α appears particularly to affect parvalbumin-expressing (PV) interneurons (8): the highest transcriptomic counts of PGC-1α are found in this subset of cortical neurons (9); reduced PGC-1α expression (heterozygotes and homozygote knockout) leads to a loss of PV in a dose-dependent manner, without affecting the expression of other interneuronal markers, whereas overexpression of PGC-1α in cultured neurons strongly induces PV expression (10). Consistent with these findings, PV interneurons ordinarily have a very high mitochondrial content and express cytochrome c more intensely than other cortical neurons (11, 12). PV interneurons are also among the most active of any cortical neuronal class; they characteristically fire on every cycle of γ oscillations (13–15) and show extremely high firing rates ahead of an ictal wave front (16, 17). These various considerations, their apparent high metabolism and the powerful effect that PV interneurons exert upon cortical networks, have led to the suggestion that PV interneurons may be particularly susceptible to metabolic stress, which in turn could give rise to neuropathology (18, 19). It is relevant, therefore, that reduced PGC-1α expression has been found in a number of neurological conditions (4), including Parkinson’s disease (20), Alzheimer’s disease (21, 22), Huntington’s disease (23), multiple sclerosis (24), and schizophrenia (25, 26), but interestingly, not epilepsy. Germline knockdown of PGC-1α is associated with hyperactive behavior (27), although this is believed to be a behavioral adaptation to a reduction in thermogenesis capacity in brown fat and muscle, because the hyperactive phenotype was not replicated in mice with central nervous system deletion of PGC-1α (28).

Knocking-out PGC-1α selectively in PV interneurons (PGC-1α^PV−/−^) also affects synaptic function, with a marked drop in two synaptic proteins, synaptotagmin 2 (SYT2) and complexin 1 (CPLX1), a reduction in synchronous GABA release, and impaired long-term memory (29). Intriguingly, the effects on GABAergic synaptic function were somewhat inconsistent in hippocampal and neocortical networks (10, 29, 30). These changes might be expected to alter network excitability, so we investigated how epileptiform activity develops in brain slices prepared from these mice compared with age-matched wild-type controls, when bathed in artificial cerebrospinal fluid (aCSF) lacking Mg^2+^ ions (0 Mg^2+^ model). Both experimental groups showed preictal activity occurring at similar rates, but the intensity of these discharges, as measured by the γ bandwidth power, and the mean firing rates in both PV and pyramidal neurons, was lower in the brain slices prepared from PGC-1α^PV−/−^ mice. Perhaps reflecting this lower intensity of discharge, the knockout brain slices experienced many more preictal events before the first seizure-like event (SLE), and consequently, also showed a significantly longer latency until this first SLE. Altogether, these data indicate that lack of PGC-1α within inhibitory PV cells acts as a restraint on runaway excitation within cortical networks having a neuroprotective effect during induced epileptogenesis. This result demonstrates a role for PGC-1α in influencing cortical excitability through transcriptional regulation of PV cell activity.

## METHODS

### Animals

All procedures were performed according to the guidelines of the Home Office UK and Animals (Scientific Procedures) Act 1986 and approved by the Newcastle University Animal Welfare and Ethical Review Body (AWERB #545). All the data were collected from adult male and female mice (2–6 mo). Wild-type (C57/BL6) mice or PVCreHet mice (Stock #008069, The Jackson Laboratory, Bar Harbor, ME) were used as control animals for all experiments. No difference was observed in the 0 Mg^2+^-induced activity between wild-type and PVCreHet mice, so these groups were combined and labeled “WT” in the study. Conditional deletion of PGC-1α within PV interneurons was achieved by crossing mice with LoxP sites flanking the exon 3–5 region of the *Ppargc1α* gene (PGC-1α^fl/fl^, Stock #009666, The Jackson Laboratory) with mice expressing Cre recombinase driven by *Pvalb* promoter (mentioned above) on a C57BL/6 background on which they were maintained. Briefly, a line of homozygous PGC-1α^fl/fl^ mice was created and crossed with a homozygous PVCre mouse line to generate the experimental animals with the genotype PVCre:PGC-1α^fl/+^. All animals were group housed in individually ventilated cages kept at room temperature with a 12 h/12 h light/dark cycle and provided with food and water ad libitum. In all experiments, data were collected from both male and female mice.

### Viral Injections

To allow visualization of PV interneurons for the patch-clamp experiments, 2- to 4-mo-old PVCre or PGC-1α.PVCre animals were injected with AAV9.Floxed.MCherry viral vector (Penn Vector Core, University of Pennsylvania). Mice were anesthetized by intraperitoneal injection of ketamine (75 mg kg^−1^, Ketalar Injection, Pfizer Ltd., Sandwich, UK) combined with medetomidine (1 mg kg^−1^, Domitor, Janssen Animal Health, Basingstoke, UK) and were then transferred to a stereotaxic frame where anesthesia was maintained using inhalational administration of isofluorane (3%–5% for induction and 2% for maintenance, delivered in O_2_ at 400–800 mL/min). The head was shaved and disinfected with chlorhexidine. A craniotomy was performed to reveal the dorsal right cortex and two injections were made of the viral vector (1 in 10 dilution) separated along the anteroposterior axis (∼1 mm and 2 mm posterior to bregma) and at the same mediolateral location (∼1.5–2 mm lateral to the midline). The viral vector was delivered by pressure injection (Ultramicropump 3, World Precision Instruments), through a 10 μL Hamilton syringe, with a beveled, 36-gauge needle (World Precision Instruments). At each site, a total of 1 µL was injected at a rate of 5–10 nL/s, at three depths (200/400/400 nL at 1/0.7/0.4 mm, respectively, deep to the pia). The needle was left in situ for 3 min, after the final injection, to prevent back-tracking of the injected particles out of the injection track. Postoperatively, mice were given subcutaneous injection of atipamezole (5 mg kg^−1^, Janssen Animal Health) and meloxicam (5 mg kg^−1^) to reverse the anesthesia and provide postoperative pain relief. The animals were allowed to recover in a heated and ventilated incubation chamber overnight and monitored at 2 and 24 h postsurgery. Some animals were given further doses of meloxicam and fluids, as required, based upon clinical assessment of their postoperative recovery, using body weight and behavioral scoring. Animals were subsequently assessed for their general health twice a day, for 5 days, and then weekly for 2–5 more wk, before being euthanized to prepare brain slices.

### Brain Slice Preparation

Mice were euthanized by cervical dislocation followed by immediate decapitation. The brain was removed and sliced into ice-cold cutting solution containing (in mM): 3 MgCl_2_; 126 NaCl; 2.6 NaHCO_3_; 3.5 KCl; 1.26 NaH_2_PO_4_; 10 glucose, using a Leica VT1200 vibratome (Nussloch, Germany). We used 400 µm horizontal sections equivalent to plates 148–158 in the Mouse Brain Atlas (31) for local field potential (LFP) recordings. Slices were then transferred to an interface tissue-holding chamber and incubated for 1–2 h at room temperature in aCSF containing (in mM): 2 CaCl_2_; 1 MgCl_2_; 126 NaCl; 2.6 NaHCO_3_; 3.5 KCl; 1.26 NaH_2_PO_4_; 10 glucose. For the patch-clamp recordings, we prepared 350-µm coronal sections, which we stored in a submerged incubation chamber for up to 4 h, before being transferred to the microscope recording chamber. All the solutions were being bubbled continuously to saturate with carboxygen (95% O_2_ and 5% CO_2_).

### Extracellular LFP Recordings

These experiments were performed using interface recording chambers. Slices were placed in the recording chamber perfused with aCSF or aCSF without Mg^2+^. Recordings were obtained using normal aCSF-filled 1–3 MΩ borosilicate glass microelectrodes (GC120TF-10; Harvard Apparatus, Kent, UK) placed in deep layers of the temporal association cortex. Microelectrodes were pulled using Narishige electrode puller (Narishige Scientific Instruments, Tokyo, Japan). The temperature of the chamber and perfusate was maintained at 33°C –36°C using a closed circulating heater (FH16D, Grant instruments, Cambridge, UK). The solutions were perfused at the rate of 3–4 mL/min by a peristaltic pump Watson Marlow 501 U (Watson–Marlow Pumps Limited, Cornwall, UK). Waveform signals were acquired using BMA-931 biopotential amplifier (Dataq instruments, Akron), Micro 1401-3 ADC board (Cambridge Electronic Design, UK), and Spike2 v. 7.10 software (Cambridge Electronic Design, UK). Signals were sampled at 10 kHz, amplified (gain: 500) and bandpass filtered (1–3,000 Hz). CED4001-16 Mains Pulser (Cambridge Electronic Design, UK) was connected to the events input of CED micro 1401-3 ADC board and was used to remove 50 Hz hum offline. Extracellular field recordings were analyzed to detect pathological discharges using a custom-written code in MATLAB2018b (The MathWorks, MA), which can be provided upon request. In brief, this used a frequency-domain analysis to detect periods when the LFP power exceeded baseline activity by a designated threshold. Using the spectrogram.m macro, in MATLAB (bin width 0.128 s, with 50% time shifts), we derived the mean and standard deviation for an epoch of baseline lasting at least 10 s, for frequencies between 1 and 150 Hz (50 subdivisions, on a log scale). The spectrogram was then performed for the entire recording and normalized to the mean and standard deviation for each frequency bandwidth individually, within this baseline epoch. To avoid contamination with mains noise, we omitted frequencies between 48 and 52 Hz. This process generated a *z*-score spectrogram, in which the pixels represented the excess power at a particular frequency, for a specific time bin (0.128 s), in terms of standard deviations from the baseline mean. Using a threshold of three standard deviations above the baseline mean, we identified all-time bins for which any frequency exceeded this threshold. These were summed cumulatively to estimate the rate and acceleration of pathological activity within the brain slice. Continuous epochs of suprathreshold bins were designated to be single “events,” which were then analyzed for their total power within the conventional LFP frequency bins, defined as follows: delta, δ, 1–4 Hz; theta, θ, 4–8 Hz; alpha, α, 8–13 Hz; beta, β, 13–30 Hz; gamma, γ, 30–80 Hz. To pool data from different brain slices, the power was normalized by power range between baseline and the full ictal activity. This was done for each bandwidth, independently, by subtracting the baseline power and then dividing by the 95th percentile range of values across the entire recording. Note that there was no systematic difference between the two groups for the baseline epochs.

Terminology: The durations of these events showed an extremely bimodal distribution, with the vast majority being less than a second duration, and a second group being long-lasting (>10 s) and which were termed “seizure-like” events. The large group of short-lasting events were termed “preictal” if they came before the first SLE or “interictal” if they came later. The durations of each event were determined as the difference between the earliest and latest point when the signal deviated >3 SD from the baseline.

### Patch-Clamp Recordings

These recordings were performed in submerged recording chambers. Slices were perfused with aCSF with the same composition, as mentioned above at 3–5 mL/min and heated to 33°C–34°C. Epileptiform activity develops in these slices in the same qualitative pattern, as seen in the interface chambers: progressively more intense preictal discharges, followed by repeated SLEs interspersed with interictal events, and finally a transition into a pattern of late-recurrent discharges. This progression occurs at a different rate, so these recordings were not used for quantification of the network activity, and only used for analyzing the firing patterns of targeted neurons. Whole cell and cell-attached data were acquired using pClamp software, Multiclamp 700B, and Digidata acquisition board (Molecular Devices, Sunnyvale, CA). Recording chamber, heater plate (Warner Instruments, Hamden, CT), and micromanipulators (Scientifica, UK) were mounted on a Scientifica movable top plate fitted to a laser spinning disk-confocal microscope (Visitech, UK). Whole cell and cell-attached recordings of layer 5 neurons within neocortex were made using 4–7 MΩ pipettes (GC150F-10, Harvard apparatus, Kent) pulled using micropipette puller (Model-P87, Sutter Instruments, CA). Pipettes were filled with solution containing (in mM): 125 K-gluconate, 6 NaCl, 10 HEPES, 2.5 Mg‐ATP, 0.3 Na_2_‐GTP; pH and osmolarity of the electrode-filling solution used were adjusted to 7.4 and 280–285 mosmol/kgH_2_O, respectively.

To investigate the firing properties of pyramidal cells and PV interneurons during induced epileptogenesis dual recordings were made in neocortical layer 5 cells in voltage-clamp configuration. First, a pyramidal cell was patched in whole cell mode and held at −70 mV to act as a reference for the development of ictal-like events in the slice and second either a pyramidal or PV cell was patched in cell-attached mode to identify spikes during evolving epileptiform activity, as monitored by the whole cell reference recording. Once the whole cell and cell-attached patch recordings were established, the Mg^2+^ ions were washed out of the bathing aCSF to induce the development of epileptiform activity. For the targeted cell-attached patch recordings of PV interneurons, the cells were visualized using a spinning disk confocal microscope (UMPlanFL *N* × 20, 0.5 NA objective) with a rhodamine (U-MRFPHQ) filter set. The tissue was illuminated with a 561-nm laser (Cobolt Jive 50; Cobolt) to visualize the mCherry fluorescence within the PV interneurons of the injected PVCre and PGC-1α.PVCre animals. Hamamatsu C9100 EM cameras (Hamamatsu Photonics (UK) Welwyn Garden City, UK) were used to visualize and capture images, run by Simple PCI software (Digital Pixel, Brighton, UK; microscope 1) installed on a Dell Precision computer (Dell Technologies, Round Rock, TX). Patch-clamp data were analyzed using custom‐written codes in Matlab2018b.

### Tissue Collection

After 1 h of recordings using the interface chamber, tissue was immersed in Ambion RNAlater solution (Thermo Fisher Scientific) and the neocortical tissue was collected. Tissue was then stored in RNAlater solution, at 4°C until the RNA was extracted.

### RNA Preparations

RNA was extracted using the RNAqueous-Micro Total RNA Isolation Kit (Thermo Fisher Scientific) and eluted in 15 µL of provided elution buffer. Residual genomic DNA was removed using reagents supplied in the above kit and done according to the manufacturer’s instructions. Concentration and purity of RNA were determined using a BioDrop DUO (BioDrop, Cambridge, UK). RNA was then stored at −80°C until the cDNA library preparation.

### Real-Time PCR

Samples were normalized to 100 ng before the cDNA library preparation. cDNA synthesis was performed using the Applied Biosystems High-Capacity cDNA Reverse Transcription Kit (Thermo Fisher Scientific) in a Thermo Hybaid PCR Express Thermal Cycler (Thermo Hybaid, Ashford, UK) at a total volume of 20 µL. The cDNA was then diluted 1:4, with nuclease-free H_2_O. The following primers were used for the real-time (RT)-PCR: Parvalbumin (For: attgaggaggatgagctggg, Rev: cgagaaggactgagatgggg), c-fos (For: cagcctttcctactaccattcc, Rev: acagatctgcgcaaaagtcc), Glyceraldehyde 3-phosphate dehydrogenase (Gapdh) (For: acctttgatgctggggctggc, Rev: gggctgagttgggatggggact), Ppargc1α (For: agcctctttgcccagatctt, Rev: ggcaatccgtcttcatccac), Syt2 (For: caactcccattcctgaccct, Rev: ttttctccaccgcccatttg), Cplx1 (For: tcagacacacttggagtccc, Rev: cacttgattatgcggccctc). Q-PCR amplifications were performed on a Applied Biosystems Step One Plus RT-PCR system (Thermo Fisher Scientific) at 95°C for 10 min, 40 repeats of 95°C for 10 s, followed by 58°C for 30 s, 95°C for 15 s. This protocol was immediately followed by the melt curve starting at 95°C for 15 s, 60°C for 1 min, 95°C for 15 s, and, finally, held at 4°C. All samples were run in duplicate at a primer molarity of 10 μmol/L, and *Pvalb*, *Fos*, *Ppargc1*α, *Syt2, Cplx1* were compared with *Gapdh*. Expression of *Gapdh* was unchanged across all treatment groups. Cycle threshold (Ct) values were analyzed using the comparative Ct method to calculate differences in gene expression between samples. Samples were run on a DNA agarose gel to confirm expected product size.

### Statistical Analysis

Relative mRNA fold changes were analyzed using the comparative Ct method. Due to a lack of homogeneity between groups (Brown–Forsythe test and Bartlett’s test), mRNA expression data were first transformed using a square root transformation. Square root transformation of mRNA data resulted in a nonsignificant Brown–Forsythe test and Bartlett’s test. Transformed data were then analyzed using a one-way analysis of variance (ANOVA), with a Tukey post hoc test for all mRNA expression data of three or more groups. Electrophysiology event detection data and mRNA of only two groups were analyzed using the Student’s *t* test, or in cases where the assumption of normality could not be made, using the Wilcoxon signed-rank test (ranksum, MATLAB). Comparisons of the γ /δ ratio (Fig. 4) were made in two ways: *1*) Wilcoxon rank test of the means (and separately the medians) for the individual recordings in the two experimental groups; *2*) Using bootstrapping resampling [in MATLAB: bootstrp(10000, @mean, datasets)] of the various datasets to estimate for each the standard error of the mean for each experimental group (32). A Gaussian fit of the probability density function yields an estimate of the standard error of the means, from which we calculated the *z*-score for the parametric differences (see Fig. 4F). Significance was set at *P* ≤ 0.05 for all analyses. Figures of mRNA expression and event detection, along with all statistics were done using GraphPad Prism (GraphPad Software, Inc., La Jolla, CA). Figures of electrophysiology traces were created using MATLAB R2018b (The MathWorks), Inkscape, and Adobe Photoshop.

## RESULTS

**Figure 1.**
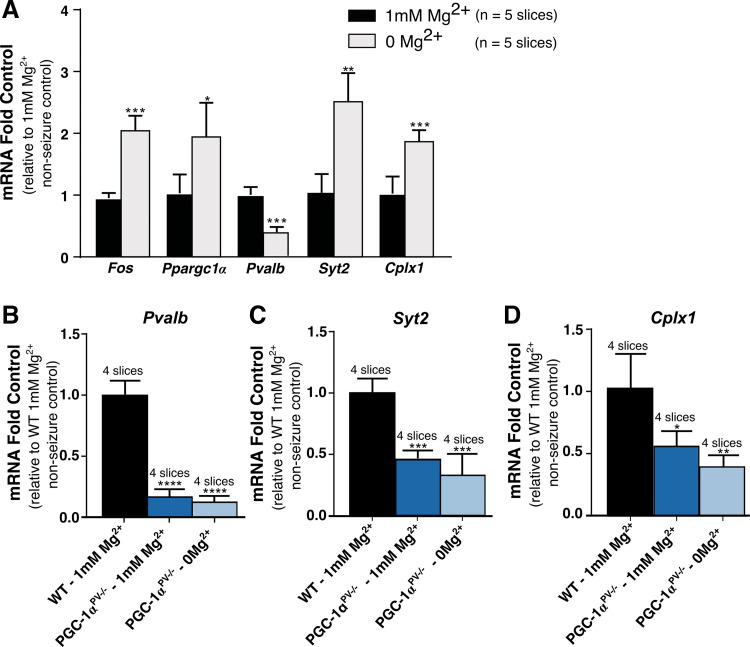
Ictogenesis-induced gene-expression changes are markedly altered in PGC-1α^PV−/−^ mice. *A*: the increased expression of *Fos*, Ppargc*1α*, parvalbumin (*Pvalb*), synaptotagmin 2 (*Syt2*), and complexin 1 (*Cplx1*) after 1 h being bathed in 0 mM Mg^2+^ artificial cerebrospinal fluid (aCSF), relative to nonseizure control (1 mM Mg^2+^). mRNA levels for parvalbumin (*Pvalb*) (*B*), synaptotagmin 2 (*Syt2*) (*C*), and complexin 1 (*Cplx1*) (*D*), normalized to the mean for wild-type brain slices kept 1 h in normal 1 mM Mg^2+^ aCSF (“control,” black). The relative change in mRNA is shown also for brain slices, prepared from mice with PGC-1α knocked out in parvalbumin (PV) cells, and bathed either in normal 1 mM Mg^2+^ (dark blue) or in 0 mM Mg^2+^ aCSF (light blue).*P < 0.05; **P < 0.01; ***P < 0.001; ****P < 0.0001.

Seizure activity rapidly induces a strong expression of protooncogenes, including c-fos. This has been demonstrated in a range of in vivo models of ictogenesis (33–35). Similarly, in brain slices, following an hour of exposure to 0 Mg^2+^ aCSF, *Fos* and *Ppargc1α* are strongly upregulated, as are the synaptic proteins, synaptotagmin 2 (*Syt2*), and complexin 1 (*Cplx1*), whereas *Pvalb* is significantly downregulated (Fig. 1*A*). The relevance of these changes in *Syt2*, *Cplx1*, and *Pvalb* is that expression of all three have previously been shown to be influenced by PGC-1α levels (10, 29). In PGC-1α^PV−/−^ brain slices, *Pvalb*, *Syt2*, and *Cplx1* all show greatly reduced expression in the naïve slices (preexposure to 0 Mg^2+^ aCSF) compared with wild-type slices, and critically, showed no alteration of expression following exposure to 0 Mg^2+^ aCSF (Fig. 1, *B*–*D*), indicating that the activity-induced changes in these genes in wild-type slices are indeed downstream of PGC-1α.

**Figure 2.**
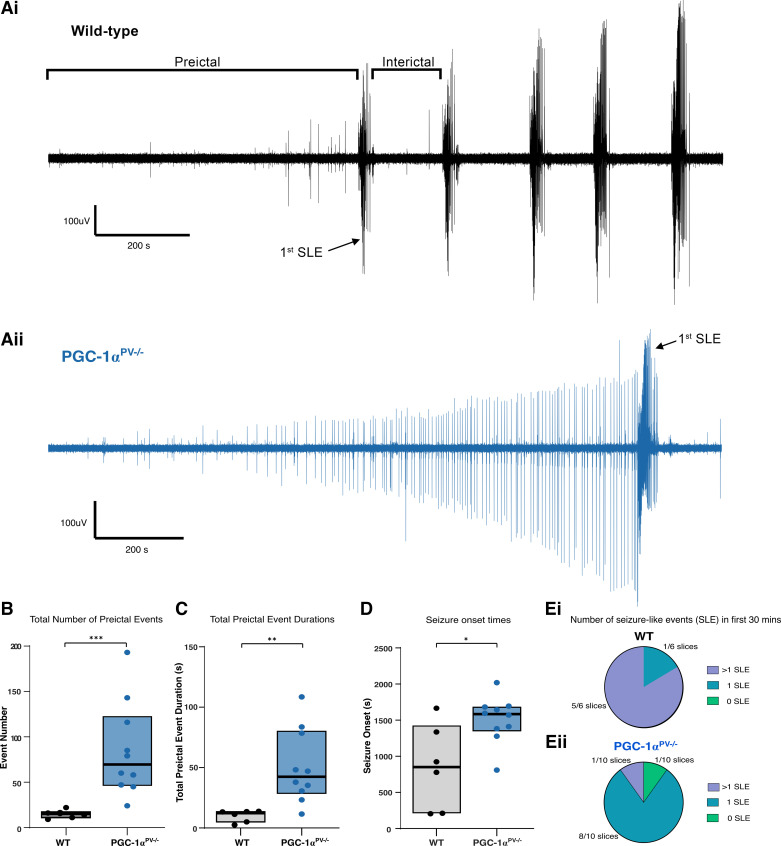
Markedly different patterns of epileptiform activity leading up to the first seizure-like event (SLE), in brain slices prepared from PGC-1α and wild-type (WT) mice. *A*: representative local field potential (LFP) recordings of activity within temporal association cortex, during 0 Mg^2+^-induced ictogenesis in brain slices prepared either from wild-type (*Ai*) or PGC-1α^PV−/−^ mice (*Aii*). *B*: boxplot showing the total number of preictal events [events before first seizure-like event (SLE; black line = median; boxed region = interquartile range)] for wild-type (black, *n* = 6 brain slices, 6 mice) and PGC-1α^PV−/−^ mice (blue, *n* = 10 slices, 10 mice). *C*: boxplot showing the total cumulative duration of pathological preictal activity. *D*: boxplot showing the latency to the first SLE. *E*: pie charts showing the number of SLEs in the first 30 min in 0 Mg^2+^ for wild-type (*Ei*) and PGC-1α^PV−/−^ mice (*Eii*). No SLEs (green), one SLE (turquoise), and more than one SLE (purple). *P < 0.05; **P < 0.01; ***P < 0.001.

**Figure 3.**
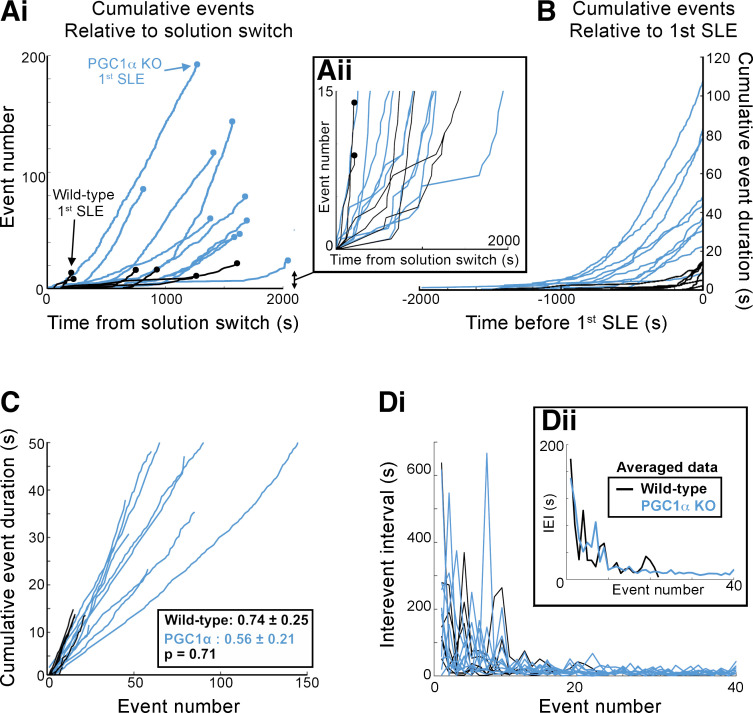
PGC-1α^PV−/−^ mice show far greater cumulative pathological activity before the onset of seizure-like events (SLEs) compared with wild-type mice. *Ai*: the cumulative number of interictal events in each brain slice, plotted relative to the time of the solution switch to 0 Mg^2+^ (black, wild-type; blue, PGC-1α^PV−/−^). The first SLE, in each case, is denoted as a terminal circle in each line. *Inset*, *Aii*: expanded view of the earliest events, note the interleaved black and blue lines. *B*: the cumulative duration of the preictal pathological activity, plotted relative to the time of onset of the first seizure-like event (SLE, designated *t* = 0 s). *C*: the same data plotted vs. the event number. Note the linearity of the cumulative plots, indicative that the event durations do not change duration over time (mean gradient ± SD), and the equivalence between the two experimental groups (Wilcoxon rank sum, *P* = 0.31). *D*: the interevent intervals (IEI), for these preictal events, plotted for each recording, with respect to the event number, for the first 40 events (this could be extended beyond 40 for the PGC-1α^PV−/−^ brain slices, but all the wild-type brain slices had had SLEs by the 22nd event. *Inset*: the means for the two experimental groups. Note how the rate settles over the first 20 events, but this happens at almost identical rates in the two experimental groups.

The lack of change in gene expression in PGC-1α^PV−/−^ brain slices was not due to a lack of epileptiform activity during the hour exposure to 0 Mg^2+^ aCSF; indeed, both sets of brain slices showed the distinctive pattern of evolving pathological discharges (Fig. 2*A*), starting with transient discharges lasting a few hundred milliseconds, and at longer latency, the appearance of sustained rhythmic discharges lasting tens of seconds, which we term seizure-like events (SLEs). The cumulative activity patterns, however, were strikingly different for the two experimental groups (Fig. 2, *B*–*E*). PGC-1α^PV−/−^ brain slices experienced a significantly larger number of preictal events before the first SLE (Fig. 2*B*, WT, *n* = 6; means ± standard deviation, 14.7 ± 4.5 preictal events per slice, with a range 9–22; PGC-1α^PV−/−^, *n* = 10; 84.6 ± 52.0 preictal events/slice, range 22–193; Wilcoxon rank sum, *P* = 0.0005) relative to control brain slices, which cumulatively represented a much longer duration of pathological activity (Fig. 2*C*). The first SLE happened significantly later in PGC-1α^PV−/−^ brain slices (Fig. 2*D*), whereas the total number of SLEs within the first 30-min exposure to 0 Mg^2+^ aCSF was significantly less compared with wild-type slices (Fig. 2*E*). Interestingly, the preictal discharges start with equivalent delay after the switch to 0 Mg^2+^ media (Fig. 3*A*); mean time to the ninth discharge (chosen because this was the start of the earliest SLE in any slice, and so represents the latest discharge that was preictal in every slice) was 645 s in wild-type brain slices (range 168–1,117 s), whereas in PGC-1α^PV−/−^ brain slices, it was 726 s (range 156–1,701 s; Wilcoxon rank-sign, *P* = 0.492, not significant). Thereafter, the cumulative duration of pathological activity rose steadily in both experimental groups, but because there were so many more preictal events in PGC-1α^PV−/−^ brain slices, the cumulative total in that experimental group achieved a far higher level before the first SLE (Fig. 3, *A* and *B*). Notably, however, the discharge durations remained stable in both groups (Fig. 3*B*; note the constant gradients). Furthermore, there was no difference in gradient between the groups. The interictal event intervals, when averaged over the entire recordings, were shorter for the PGC1α^PV−/−^ brain slices (26.8 ± 23.0 s; WT 552 ± 23.0 s), but this appears to be explained entirely by the fact that the two groups are not sampled equivalently: on average, there were 85 preictal events in PGC1α^PV−/−^ brain slices, but only 15 preictal events in WT brain slices, and the earliest events have the longest interevent intervals in both groups. If we analyze just the first 15 events in the PGC1α^PV−/−^ brain slices (IEI = 63.2 ± 34.1 s), or examine the evolving interictal event intervals over these first 20 events, the two experimental groups were almost identical (Fig. 3*D*). Collectively, these data indicate that the delay to the first SLE in PGC-1α^PV−/−^ brain slices is due to the fact that these slice tolerate a far larger cumulative load of preictal discharges before progressing to ictal levels of activation.

**Figure 4.**
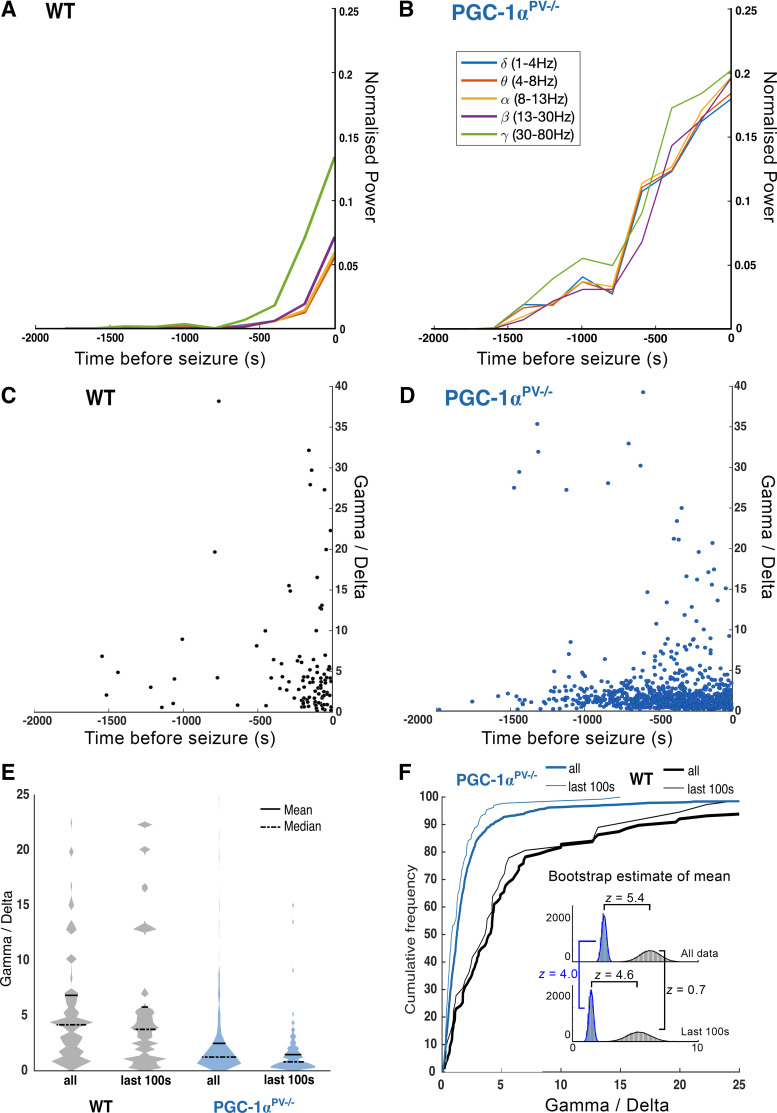
Preictal discharges in brain slices from PGC-1α knockout mice show reduced normalized gamma activity (γ/δ ratio) compared with wild-type (WT) mice. *A*: the mean normalized power, for the main physiological LFP bandwidths as indicated, averaged for sequential 200 s epochs before the onset of the first SLE (*t* = 0 s), for wild-type brain slices, recorded in 0 Mg^2+^ ACSF (*n* = 7). *B*: equivalent data from brain slices from PGC-1α^PV−/−^ mice (*n* = 10). Note the far more rapid escalation of activity in the wild-type brain slices, especially in the gamma and high-gamma frequency bands, leading up to the first SLE. The γ/δ ratio for every single interictal event, plotted against time relative to the onset of the first SLE, in the wild-type brain slices (*C*), and in the PGC-1α^PV−/−^ brain slices (*D*). *E*: violin plots of γ/δ ratios, for the entire data set of interictal events, and, events occurring in the 100 s immediately before the first SLE. *F*: cumulative frequency plots of the same data sets. *Insets*: the probability distribution for estimates of the means for the full data sets, and the events within 100 s before the first SLE, derived by bootstrap resampling with replacement. *Z*-scores are provided for the four group comparisons, all except the wild-type, all-vs.-last 100 s comparisons are highly significant.

We next analyzed the power spectra of each preictal discharge (Fig. 4), which showed that although the durations remain stable, the power across all bandwidths increased progressively, leading up to the first SLE (Fig. 4, *A* and *B*). The low-frequency component of the LFP is thought to reflect the local synaptic currents, whereas the high γ activity scales with the level of local firing. The ratio of these two bandwidths, therefore, is a measure of the degree to which synaptic volleys induce local postsynaptic firing. Note that the ratios were of the normalized bandwidths, and so this analysis examines whether one bandwidth is changing relative to another (rather than their absolute ratio). The distribution pattern of the γ /δ ratio with respect to time before the first SLE differed in two respects between the two experimental groups (Fig. 4, *C* and *D*). First, the progression toward the first SLE appears greatly delayed in the PGC-1α^PV−/−^ brain slices, relative to the wild-type controls, and so the distribution is spread across a far longer time period, with many more events in the period from 1,500 s to 250 s before the SLE (Fig. 4, *B* and *D*). The second difference was that the PGC-1α^PV−/−^ brain slices showed a compressed distribution of the γ/δ ratio, across the entire data set (Fig. 4, *E* and *F*), and similarly for the events occurring in the last 100 s before the first SLE. As the distributions were skewed, for each experimental group (Fig. 4*E*), we examined the differences in three different ways. The Wilcoxon sign-rank test performed on the medians from each brain slice showed highly significant difference between the WT and PGC-1α^PV−/−^ brain slices (*P* = 0.042), and a similar analysis based upon the means gave the same result (*P* = 0.031). We confirmed these findings also using bootstrap resampling, with replacement (32), to estimate the probability distribution of the means for each experimental group (Fig. 4*F*, *inset*). In each case, these were well fitted by a Gaussian curve, thereby yielding estimates of the standard error of the mean (all data, WT vs. PGC-1α^PV−/−^, *P* < 10^−5^; last 100 s, WT vs. PGC-1α^PV−/−^, *P* < 10^−5^; WT, all vs. last 100 s, not significant; PGC-1α^PV−/−^, all vs. last 100 s, *P* = 3 × 10^−5^). These analyses thus confirm the result shown in Fig. 4, *A* and *B*, that the γ oscillation appears to escalate excessively, in the wild-type brain slices, and that by comparison, the γ power scales up more slowly in the PGC-1α^PV−/−^ brain slices.

**Figure 5.**
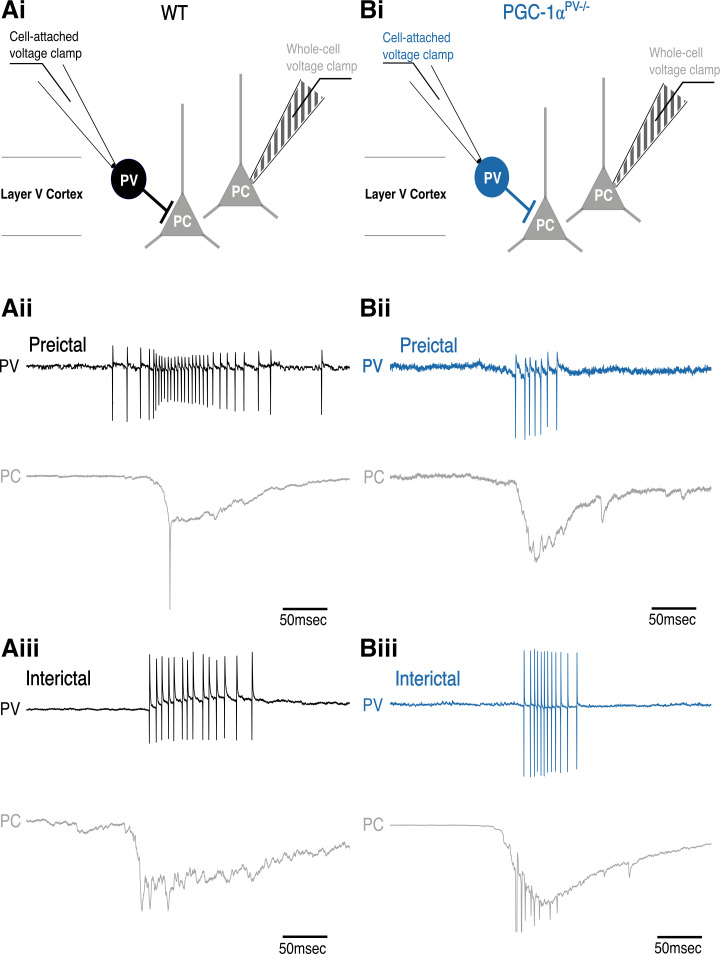
Example parvalbumin (PV) interneuron preictal and interictal events (*Ai* and *Bi*). The recording configuration for the example traces, below, with a whole cell, voltage-clamp, pyramidal cell (PC) recording (triangle and striped electrode) and a cell-attached recording of action potentials in a PV interneuron (circle and clear electrode). The blue traces are from PV interneurons in which PGC-1α is not expressed (PGC-1α^PV−/−^ mice); the black traces are from PV interneurons recorded in wild-type brain slices. The underlying panels show example pairs of recordings of “preictal” discharges (before the first SLE, *Aii*, *Bii*) and “interictal” discharges (after the first SLE, but between SLE events, *Aiii*, *Biii*). For each paired recording, the top traces show the cell-attached interneuron recording and the lower traces show the whole cell pyramidal recording.

**Figure 6.**
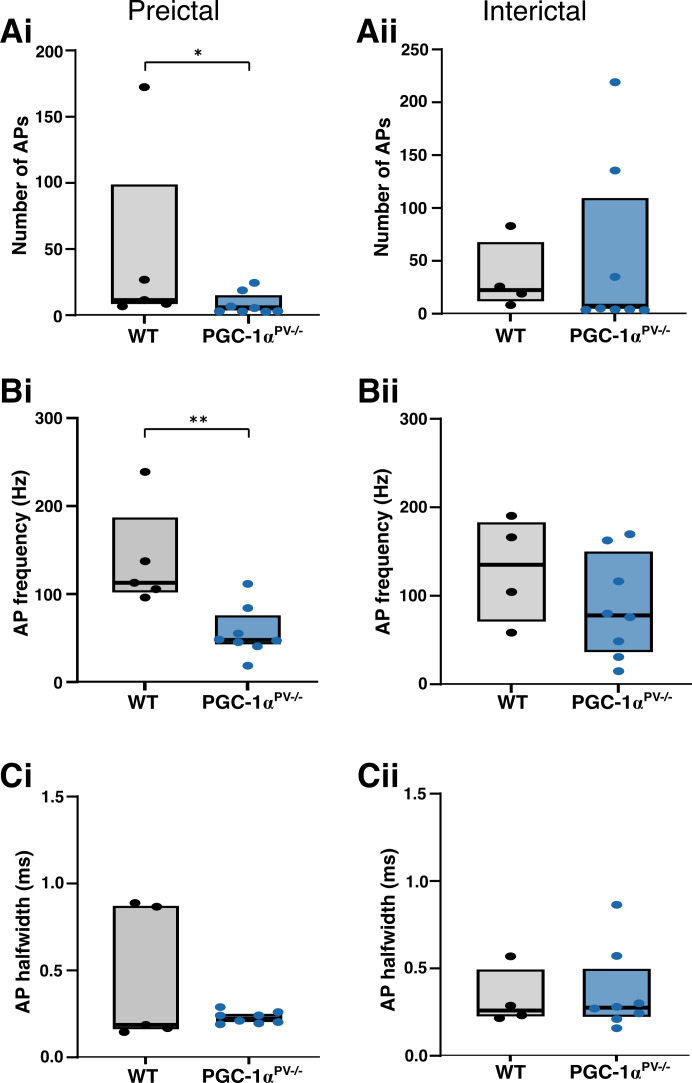
Action potential (AP) properties of PV interneurons during preictal and interictal events following 0 Mg^2+^-induced ictogenesis. This figure shows cortical PV interneuron action potential numbers (*Ai*, *Aii*, WT, *n* = 5 brain slices, from 3 mice; KO, *n* = 8, 4 mice), their frequency (*Bi*, *Bii*), and halfwidths (*Ci*, *Cii*) during preictal (*left* column) and interictal events (*right* column) induced by 0 Mg^2+^-ictogenic artificial cerebrospinal fluid (aCSF) in control (black) and PGC-1α^PV−/−^ (red) mouse tissue. Data points show the average data per brain slice with the median shown as a black line and the box indicating the interquartile range. *P < 0.05; **P < 0.01. KO, knockout; WT, wild-type.

To investigate these differences further, we made paired patch-clamp recordings from PV interneurons and pyramidal cells (Fig. 5). The pyramidal recording was made in whole cell voltage-clamp mode to record the synaptic bombardment, whereas the PV recording was made in cell-attached mode to record the firing pattern (Fig. 5, *Ai* and *Bi*). This allowed us to compare the level of PV participation in these discharges, in the wild-type and PGC-1α^PV−/−^ experimental groups. We found that PV firing rates were significantly lower in the PGC-1α^PV−/−^ brain slices compared with wild-type brain slices before the first SLE (Fig. 6*Ai*), although this difference was not maintained in later interictal events (Fig. 6*Aii*). The number of action potentials per discharge (Fig. 6*B*), and the action potential durations (Fig. 6*C*), was equivalent in the wild-type and PGC-1α^PV−/−^ brain slices.

**Figure 7.**
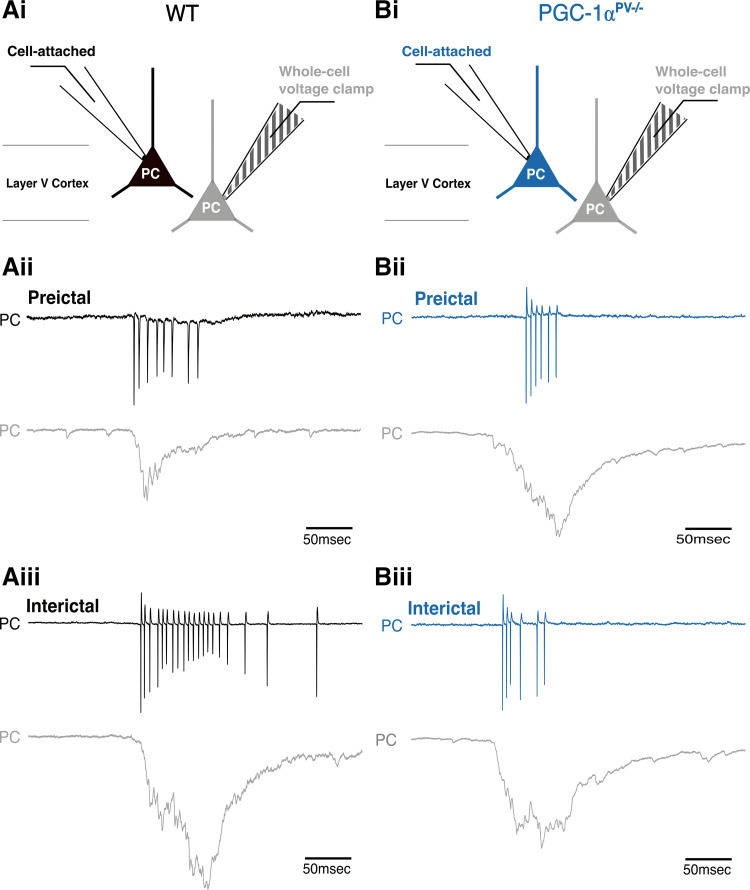
PGC-1α^PV−/−^ mice show reduced pyramidal firing rates, during preictal discharges compared with wild-type (WT) mice. *Ai* and *Bi*: the recording configuration for the underlying traces (as per Fig. 5 except recording action potentials in pyramidal cells). The blue traces indicate the action potential, cell-attached recordings from the PGC-1α^PV−/−^ brain slices (note that the recorded cells actually express PGC-1α, as the knockout is specific to the PV interneurons). The underlying panels show preictal (*Aii*, *Bii*), and interictal (*Aiii*, *Biii*) pyramidal cell events. In each pair of recordings, the top traces show the cell-attached recording (gray) and the lower traces show the whole cell recording.

**Figure 8.**
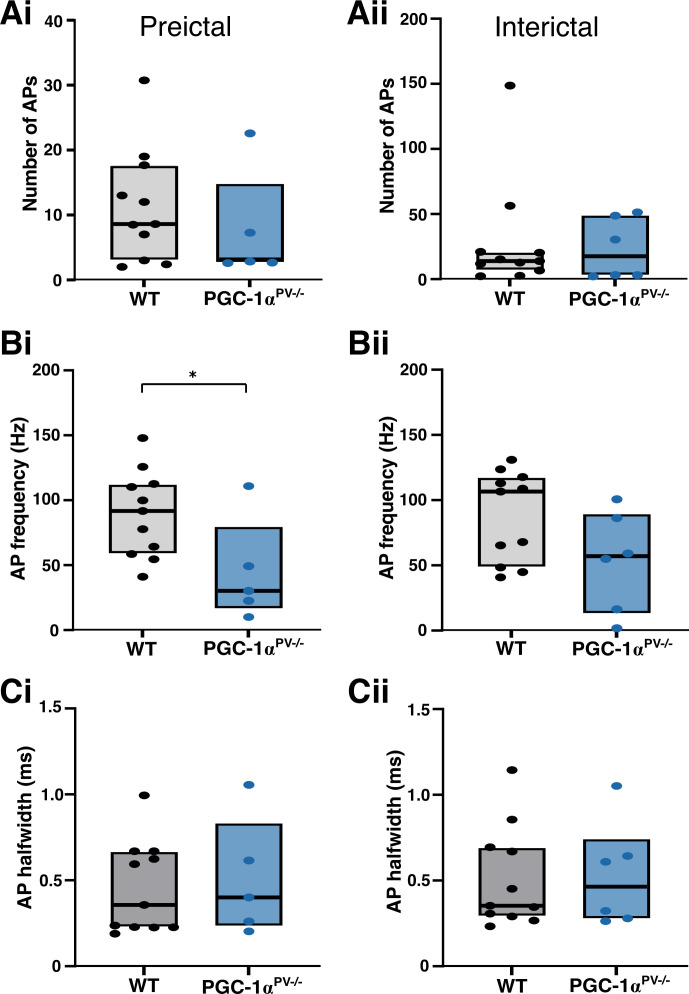
Action potential (AP) properties of pyramidal cells during preictal and interictal events following 0 Mg^2+^-induced ictogenesis. This figure shows cortical pyramidal cell action potential numbers (*Ai*, *Aii*, WT, *n* = 11 brain slices, from 6 mice; KO, *n* = 6 slices, 4 mice), their frequency (*Bi*, *Bii*) and halfwidths (*Ci*, *Cii*) during preictal (left column) and interictal events (right column) induced by 0 Mg^2+^-ictogenic artificial cerebrospinal fluid (aCSF) in control (gray/black) and PGC-1α^PV−/−^ (red) mouse tissue. Data points show the average data per brain slice, with the median shown as a black line and the box indicating the interquartile range. *P < 0.05. KO, knockout; WT, wild-type.

We then repeated the experiment, but instead, recording the firing patterns of the pyramidal cells (Fig. 7). The numbers of action potentials per discharge was similar for the two experimental groups (Fig. 8*A*), but the maximal firing rate for the PGC-1α^PV−/−^ brain slices was significantly lower than for the wild-type brain slices, consistent with the lower γ /δ ratio (Fig. 8*B*). This difference, however, was only seen for the preictal events, and not the interictal ones (after the first SLE; Fig. 8*B*). There was no difference in the half-width of the action potentials in the two groups (Fig. 8*C*). These analyses show, therefore, that even though the genetic manipulation was restricted to the PV interneuronal cell class, the network impact was felt more widely across the network.

## DISCUSSION

We have shown in these studies that knocking down the transcriptional coactivator, PGC-1α, specifically in PV interneurons, reduces network excitability. Our focus upon this subpopulation of interneurons was motivated in part because they have been postulated to play pivotal roles in various physiological brain rhythms (36–38), as well as both opposing (16, 39), and promoting (40), seizure-like activity in different situations (note that these two statements are not necessarily incompatible, see Refs. 17 and 41–43). Finally, there is a close relationship between PGC-1α and PV expression (10), for which we provide further evidence by showing that activity dependent regulation of genes downstream of PGC-1α, such as *Pvalb*, is lost in the knockout (Fig. 1, *B*–*D*). The antiepileptic effect reflects a reduction in the intensity of preictal bursts, measured both by the high γ power, and also by the firing rates of both PV interneurons and pyramidal cells.

The effect on PV interneuronal firing could be understood as a direct consequence of the fact that these are the cells in which PGC-1α is knocked down. As such, it could be interpreted as an adaptation to a mutation that limits energy production, by limiting also the energy expenditure. However, the secondary effect on pyramidal firing tells us that there are indirect consequences of the cell-specific knockdown, and given that interneuronal firing is driven by pyramidal activity, there are clearly more complex feedback loops that cloud any simple interpretation of the results. This is further complicated by other effects, such as the reduction in expression of genes downstream of PGC-1α; synaptotagmin 2 (*Syt2*) and complexin 1 (*Cplx1*). SYT2 and CPLX1 are synaptic proteins which act to promote synchronous neurotransmitter release from PV cells and their expression levels are reduced in PGC-1α^PV−/−^ mice (29). We confirmed this reduction and additionally showed a lack of activity-dependent regulation of expression levels, which resulted in a failure to increase expression in response to induced hyperexcitability.

Another interesting element is the reduction also in PV expression in PGC-1α^PV−/−^ mice. PV acts as a slow calcium buffer, helping to protect against hyperexcitability by sequestering away excess Ca^2+^ during periods of intense activity. Altered Ca^2+^ buffering in PGC-1α^PV−/−^ mice may therefore be a factor in the reduced level of interneuronal bursting, although it is notable that the specific knockdown of PV in striatal interneurons is associated with increased excitability (44), the opposite of what we found during induced ictogenesis. The different results may arise for several reasons: our studies involved a reduction in PV expression, rather than a complete PV deficiency; they were of neocortical, rather than striatal interneurons; and the activity patterns and input to PV cells during 0 Mg^2+^-induced ictogenesis may differ substantially from those of electrical stimulation paradigms, and as already mentioned, are influenced by multiple other factors besides.

The nonautonomous cellular effect seen in pyramidal cells may also arise through a complex set of interactions. One possibility is suggested by the finding that γ activity rises disproportionately relative to the increases in δ activity, in the wild-type experimental group (see Fig. 4*A*) compared with the PGC-1α^PV−/−^ brain slices (Fig. 4*B*). The rate of escalation in epileptiform activity can be explained in terms of the rate of chloride loading, which in turn, depends on the rate of PV firing. Lowering the PV activity, as is seen in the PGC-1α^PV−/−^ brain slices, would reduce this effect, slowing the escalation, and when averaged over multiple preictal events, yields a lower mean discharge rate also for the pyramidal population. In contrast, the intense GABAergic discharges seen in wild-type brain slices, while serving a protective purpose also has the unwanted consequence of loading their postsynaptic target cells with chloride (45), thereby contributing to the pathological evolution of activity (41–43, 46–49). This is an example of positive feedback, where raised network activity creates the substrate that facilitates further neuronal activation that may be critical for seizure initiation (50).

Early studies of PGC-1α identified its thermogenic role in skeletal muscle and brown fat, by uncoupling mitochondrial respiration in response to cold (51); interestingly, cold does not induce PGC-1α in the brain. There, its role may relate to protection against other stresses, including cytokines and lipopolysaccharides (4, 5). For instance, PGC-1α leads to increases in the expression of various enzymes involved in detoxification of reactive oxygen species (1). Conversely, in mouse models of stress, PGC-1α reduction associated with increased markers of oxidative stress are found within cortical PV interneurons (52). The cellular mechanism appears to involve activation of the p38 MAPK pathway, leading to phosphorylation of PGC-1α, which stabilizes the protein and increases its transcription-inducing activity (5). It is noteworthy, therefore, that inhibition of the p38 MAPK pathway has also been reported to increase the latency to the first seizure, following pilocarpine treatment (53). That is to say, the pharmacological blockade of MAPK and the knockdown of one of its targets, PGC-1α, as we show in this study, appear to have similar effects, both slowing the pathological process in acute models of ictogenesis. Inhibiting p38 MAPK also reduces the frequency of seizures, and the level of associated pathological damage within the hippocampus (53). In contrast, periods of status epilepticus activate p38 MAPK and this may mediate the subsequent anatomical changes within the cortical networks (54).

Reduced PGC-1α has been associated with a range of neurological conditions (20–26), but not, to date, with epilepsy. The most pertinent of these to our study is the association with schizophrenia (25, 26), for which the pathophysiological role of PGC-1α is also believed to be manifest through the PV interneurons within cortical networks (26, 55). The clinical association between epilepsy and schizophrenia is well established, with the existence of one condition carrying around a 6- to 8-fold increase in risk for having the other (56). This, though presents something of a paradox, as PV-specific knockout of PGC-1α in mice has opposite effects, by increasing the likelihood of a schizophrenic phenotype, while reducing the epileptic phenotype. The answer may lie in our finding that the PV-specific deletion of PGC-1α appears to allow more preictal pathological discharges and this may be the source of schizophrenic pathophysiology. Previous work reported that knocking down PGC-1α in PV interneurons alters the asynchronous synaptic release of GABA (29), which may be expected to alter how PV interneurons influence spike timing and information flow through cortical networks. Our work suggests that different forms of PV interneuron pathophysiology, relevant to epilepsy and schizophrenia, are distinguished by the level of involvement of PGC-1α.

## GRANTS

This work was supported by a Wellcome Trust-NIH PhD studentship 205944/Z/17/Z (to C.M.-G.S.) and grants from Epilepsy Research UK (P1504), BBSRC (BB/P019854/1), and MRC (MR/R005427/1).

## DISCLOSURES

No conflicts of interest, financial or otherwise, are declared by the authors. 

## AUTHOR CONTRIBUTIONS

R.R.P. and A.J.T. conceived and designed research; C.M.-G.S. and R.R.P. performed experiments; C.M.-G.S., R.R.P., and D.W. analyzed data; C.M.-G.S., R.R.P., and A.J.T. interpreted results of experiments; C.M.-G.S., R.R.P., D.W., and A.J.T. prepared figures; A.J.T. and C.M.-G.S. drafted manuscript; C.M.-G.S., R.R.P., D.W., C.R., R.M.C., and A.J.T. edited and revised manuscript; C.M.-G.S., R.R.P., D.W., C.R., R.M.C., and A.J.T. approved final version of manuscript. 

## References

[1] Austin S, St-Pierre J. PGC1α and mitochondrial metabolism—emerging concepts and relevance in ageing and neurodegenerative disorders. J Cell Sci 125: 4963–4971, 2012. doi:10.1242/jcs.113662. 23277535

[2] Handschin C, Spiegelman BM. Peroxisome proliferator-activated receptor gamma coactivator 1 coactivators, energy homeostasis, and metabolism. Endocr Rev 27: 728–735, 2006. doi:10.1210/er.2006-0037. 17018837

[3] Lin J, Handschin C, Spiegelman BM. Metabolic control through the PGC-1 family of transcription coactivators. Cell Metab 1: 361–370, 2005. doi:10.1016/j.cmet.2005.05.004. 16054085

[4] McMeekin LJ, Fox SN, Boas SM, Cowell RM. Dysregulation of PGC-1α-dependent transcriptional programs in neurological and developmental disorders: therapeutic challenges and opportunities. Cells 10: 352, 2021. doi:10.3390/cells10020352. 33572179 PMC7915819

[5] Puigserver P, Rhee J, Lin J, Wu Z, Yoon JC, Zhang CY, Krauss S, Mootha VK, Lowell BB, Spiegelman BM. Cytokine stimulation of energy expenditure through p38 MAP kinase activation of PPARγ coactivator-1. Mol Cell 8: 971–982, 2001. doi:10.1016/S1097-2765(01)00390-2. 11741533

[6] Bagattin A, Hugendubler L, Mueller E. Transcriptional coactivator PGC-1α promotes peroxisomal remodeling and biogenesis. Proc Natl Acad Sci USA 107: 20376–20381, 2010. doi:10.1073/pnas.1009176107. 21059926 PMC2996647

[7] St-Pierre J, Lin J, Krauss S, Tarr PT, Yang R, Newgard CB, Spiegelman BM. Bioenergetic analysis of peroxisome proliferator-activated receptor γ coactivators 1α and 1β (PGC-1α and PGC-1β) in muscle cells. J Biol Chem 278: 26597–26603, 2003. doi:10.1074/jbc.M301850200. 12734177

[8] Cowell RM, Blake KR, Russell JW. Localization of the transcriptional coactivator PGC-1α to GABAergic neurons during maturation of the rat brain. J Comp Neurol 502: 1–18, 2007. doi:10.1002/cne.21211. 17335037

[9] Saunders A, Macosko EZ, Wysoker A, Goldman M, Krienen FM, de Rivera H, Bien E, Baum M, Bortolin L, Wang S, Goeva A, Nemesh J, Kamitaki N, Brumbaugh S, Kulp D, McCarroll SA. Molecular diversity and specializations among the cells of the adult mouse brain. Cell 174: 1015–1030.e16, 2018. doi:10.1016/j.cell.2018.07.028. 30096299 PMC6447408

[10] Lucas EK, Markwardt SJ, Gupta S, Meador-Woodruff JH, Lin JD, Overstreet-Wadiche L, Cowell RM. Parvalbumin deficiency and GABAergic dysfunction in mice lacking PGC-1α. J Neurosci 30: 7227–7235, 2010. doi:10.1523/JNEUROSCI.0698-10.2010. 20505089 PMC2888101

[11] Gulyás AI, Buzsáki G, Freund TF, Hirase H. Populations of hippocampal inhibitory neurons express different levels of cytochrome c. Eur J Neurosci 23: 2581–2594, 2006. doi:10.1111/j.1460-9568.2006.04814.x. 16817861

[12] Kageyama GH, Wong-Riley MT. Histochemical localization of cytochrome oxidase in the hippocampus: correlation with specific neuronal types and afferent pathways. Neuroscience 7: 2337–2361, 1982. doi:10.1016/0306-4522(82)90199-3. 6294558

[13] Atallah BV, Scanziani M. Instantaneous modulation of gamma oscillation frequency by balancing excitation with inhibition. Neuron 62: 566–577, 2009. doi:10.1016/j.neuron.2009.04.027. 19477157 PMC2702525

[14] Hájos N, Pálhalmi J, Mann EO, Németh B, Paulsen O, Freund TF. Spike timing of distinct types of GABAergic interneuron during hippocampal gamma oscillations in vitro. J Neurosci 24: 9127–9137, 2004. doi:10.1523/JNEUROSCI.2113-04.2004. 15483131 PMC6730063

[15] Whittington MA, Traub RD. Inhibitory interneurons and network oscillations in vitro. Trends Neurosci 26: 676–682, 2003. doi:10.1016/j.tins.2003.09.016. 14624852

[16] Parrish RR, Codadu NK, Mackenzie-Gray Scott C, Trevelyan AJ. Feedforward inhibition ahead of ictal wavefronts is provided by both parvalbumin- and somatostatin-expressing interneurons. J Physiol 597: 2297–2314, 2019. doi:10.1113/JP277749. 30784081 PMC6462485

[17] Sessolo M, Marcon I, Bovetti S, Losi G, Cammarota M, Ratto GM, Fellin T, Carmignoto G. Parvalbumin-positive inhibitory interneurons oppose propagation but favor generation of focal epileptiform activity. J Neurosci 35: 9544–9557, 2015. doi:10.1523/JNEUROSCI.5117-14.2015. 26134638 PMC6605139

[18] Kann O. The interneuron energy hypothesis: implications for brain disease. Neurobiol Dis 90: 75–85, 2016. doi:10.1016/j.nbd.2015.08.005. 26284893

[19] Whittaker RG, Turnbull DM, Whittington MA, Cunningham MO. Impaired mitochondrial function abolishes gamma oscillations in the hippocampus through an effect on fast-spiking interneurons. Brain 134: e180, 2011. doi:10.1093/brain/awr018. 21378098 PMC3708717

[20] Zheng B, Liao Z, Locascio JJ, Lesniak KA, Roderick SS, Watt ML, Eklund AC, Zhang-James Y, Kim PD, Hauser MA, Grünblatt E, Moran LB, Mandel SA, Riederer P, Miller RM, Federoff HJ, Wüllner U, Papapetropoulos S, Youdim MB, Cantuti-Castelvetri I, Young AB, Vance JM, Davis RL, Hedreen JC, Adler CH, Beach TG, Graeber MB, Middleton FA, Rochet J-C, Scherzer CR; Global PD Gene Expression (GPEX) Consortium. PGC-1α, a potential therapeutic target for early intervention in Parkinson's disease. Sci Transl Med 2: 52ra73, 2010. doi:10.1126/scitranslmed.3001059. 20926834 PMC3129986

[21] Qin W, Haroutunian V, Katsel P, Cardozo CP, Ho L, Buxbaum JD, Pasinetti GM. PGC-1α expression decreases in the Alzheimer disease brain as a function of dementia. Arch Neurol 66: 352–361, 2009. doi:10.1001/archneurol.2008.588. 19273754 PMC3052997

[22] Sheng B, Wang X, Su B, Lee HG, Casadesus G, Perry G, Zhu X. Impaired mitochondrial biogenesis contributes to mitochondrial dysfunction in Alzheimer's disease. J Neurochem 120: 419–429, 2012. doi:10.1111/j.1471-4159.2011.07581.x. 22077634 PMC3253532

[23] Cui L, Jeong H, Borovecki F, Parkhurst CN, Tanese N, Krainc D. Transcriptional repression of PGC-1α by mutant huntingtin leads to mitochondrial dysfunction and neurodegeneration. Cell 127: 59–69, 2006. doi:10.1016/j.cell.2006.09.015. 17018277

[24] Witte ME, Nijland PG, Drexhage JA, Gerritsen W, Geerts D, van Het Hof B, Reijerkerk A, de Vries HE, van der Valk P, van Horssen J. Reduced expression of PGC-1α partly underlies mitochondrial changes and correlates with neuronal loss in multiple sclerosis cortex. Acta Neuropathol 125: 231–243, 2013. doi:10.1007/s00401-012-1052-y. 23073717

[25] Christoforou A, Le Hellard S, Thomson PA, Morris SW, Tenesa A, Pickard BS, Wray NR, Muir WJ, Blackwood DH, Porteous DJ, Evans KL. Association analysis of the chromosome 4p15-p16 candidate region for bipolar disorder and schizophrenia. Mol Psychiatry 12: 1011–1025, 2007. doi:10.1038/sj.mp.4002003. 17457313

[26] Jiang Z, Cowell RM, Nakazawa K. Convergence of genetic and environmental factors on parvalbumin-positive interneurons in schizophrenia. Front Behav Neurosci 7: 116, 2013. doi:10.3389/fnbeh.2013.00116. 24027504 PMC3759852

[27] Lin J, Wu PH, Tarr PT, Lindenberg KS, St-Pierre J, Zhang CY, Mootha VK, Jäger S, Vianna CR, Reznick RM, Cui L, Manieri M, Donovan MX, Wu Z, Cooper MP, Fan MC, Rohas LM, Zavacki AM, Cinti S, Shulman GI, Lowell BB, Krainc D, Spiegelman BM. Defects in adaptive energy metabolism with CNS-linked hyperactivity in PGC-1α null mice. Cell 119: 121–135, 2004. doi:10.1016/j.cell.2004.09.013. 15454086

[28] Lucas EK, Dougherty SE, McMeekin LJ, Trinh AT, Reid CS, Cowell RM. Developmental alterations in motor coordination and medium spiny neuron markers in mice lacking pgc-1α. PLoS One 7: e42878, 2012. doi:10.1371/journal.pone.0042878. 22916173 PMC3419240

[29] Lucas EK, Dougherty SE, McMeekin LJ, Reid CS, Dobrunz LE, West AB, Hablitz JJ, Cowell RM. PGC-1α provides a transcriptional framework for synchronous neurotransmitter release from parvalbumin-positive interneurons. J Neurosci 34: 14375–14387, 2014. doi:10.1523/JNEUROSCI.1222-14.2014. 25339750 PMC4205559

[30] Dougherty SE, Bartley AF, Lucas EK, Hablitz JJ, Dobrunz LE, Cowell RM. Mice lacking the transcriptional coactivator PGC-1α exhibit alterations in inhibitory synaptic transmission in the motor cortex. Neuroscience 271: 137–148, 2014. doi:10.1016/j.neuroscience.2014.04.023. 24769433 PMC4068733

[31] Franklin KBJ, Paxinos G. The Mouse Brain in Stereotaxic Coordinates (3rd ed.). Amsterdam: Academic Press, 2008.

[32] Kulesa A, Krzywinski M, Blainey P, Altman N. Sampling distributions and the bootstrap. Nat Methods 12: 477–478, 2015. doi:10.1038/nmeth.3414. 26221652 PMC4737599

[33] Dragunow M, Robertson HA. Kindling stimulation induces c-fos protein(s) in granule cells of the rat dentate gyrus. Nature 329: 441–442, 1987. doi:10.1038/329441a0. 3116433

[34] Morgan JI, Cohen DR, Hempstead JL, Curran T. Mapping patterns of c-fos expression in the central nervous system after seizure. Science 237: 192–197, 1987. doi:10.1126/science.3037702. 3037702

[35] White JD, Gall CM. Differential regulation of neuropeptide and proto-oncogene mRNA content in the hippocampus following recurrent seizures. Brain Res 427: 21–29, 1987. doi:10.1016/0169-328X(87)90040-4. 3427445

[36] Cobb SR, Buhl EH, Halasy K, Paulsen O, Somogyi P. Synchronization of neuronal activity in hippocampus by individual GABAergic interneurons. Nature 378: 75–78, 1995. doi:10.1038/378075a0. 7477292

[37] Sohal VS, Zhang F, Yizhar O, Deisseroth K. Parvalbumin neurons and gamma rhythms enhance cortical circuit performance. Nature 459: 698–702, 2009. doi:10.1038/nature07991. 19396159 PMC3969859

[38] Traub RD, Miles R. Neuronal Networks of the Hippocampus. Cambridge, UK: Cambridge University Press, 1991.

[39] Trevelyan AJ. Do cortical circuits need protecting from themselves? Trends Neurosci 39: 502–511, 2016. doi:10.1016/j.tins.2016.06.002. 27378547

[40] Chang M, Dian JA, Dufour S, Wang L, Moradi Chameh H, Ramani M, Zhang L, Carlen PL, Womelsdorf T, Valiante TA. Brief activation of GABAergic interneurons initiates the transition to ictal events through post-inhibitory rebound excitation. Neurobiol Dis 109: 102–116, 2018. doi:10.1016/j.nbd.2017.10.007. 29024712

[41] Burman RJ, Selfe JS, Lee JH, van den Berg M, Calin A, Codadu NK, Wright R, Newey SE, Parrish RR, Katz AA, Wilmshurst JM, Akerman CJ, Trevelyan AJ, Raimondo JV. Excitatory GABAergic signalling is associated with benzodiazepine resistance in status epilepticus. Brain 142: 3482–3501, 2019. doi:10.1093/brain/awz283. 31553050 PMC6904319

[42] Cossart R, Bernard C, Ben-Ari Y. Multiple facets of GABAergic neurons and synapses: multiple fates of GABA signalling in epilepsies. Trends Neurosci 28: 108–115, 2005. doi:10.1016/j.tins.2004.11.011. 15667934

[43] Ellender TJ, Raimondo JV, Irkle A, Lamsa KP, Akerman CJ. Excitatory effects of parvalbumin-expressing interneurons maintain hippocampal epileptiform activity via synchronous afterdischarges. J Neurosci 34: 15208–15222, 2014. doi:10.1523/JNEUROSCI.1747-14.2014. 25392490 PMC4228130

[44] Orduz D, Bischop DP, Schwaller B, Schiffmann SN, Gall D. Parvalbumin tunes spike-timing and efferent short-term plasticity in striatal fast spiking interneurons. J Physiol 591: 3215–3232, 2013. doi:10.1113/jphysiol.2012.250795. 23551945 PMC3717224

[45] Thompson SM, Gähwiler BH. Activity-dependent disinhibition. I. Repetitive stimulation reduces IPSP driving force and conductance in the hippocampus in vitro. J Neurophysiol 61: 501–511, 1989. doi:10.1152/jn.1989.61.3.501. 2709096

[46] Alfonsa H, Merricks EM, Codadu NK, Cunningham MO, Deisseroth K, Racca C, Trevelyan AJ. The contribution of raised intraneuronal chloride to epileptic network activity. J Neurosci 35: 7715–7726, 2015. doi:10.1523/JNEUROSCI.4105-14.2015. 25995461 PMC4438123

[47] Dzhala VI, Kuchibhotla KV, Glykys JC, Kahle KT, Swiercz WB, Feng G, Kuner T, Augustine GJ, Bacskai BJ, Staley KJ. Progressive NKCC1-dependent neuronal chloride accumulation during neonatal seizures. J Neurosci 30: 11745–11761, 2010. doi:10.1523/JNEUROSCI.1769-10.2010. 20810895 PMC3070296

[48] Huberfeld G, Wittner L, Clemenceau S, Baulac M, Kaila K, Miles R, Rivera C. Perturbed chloride homeostasis and GABAergic signaling in human temporal lobe epilepsy. J Neurosci 27: 9866–9873, 2007. doi:10.1523/JNEUROSCI.2761-07.2007. 17855601 PMC6672644

[49] Lillis KP, Kramer MA, Mertz J, Staley KJ, White JA. Pyramidal cells accumulate chloride at seizure onset. Neurobiol Dis 47: 358–366, 2012. doi:10.1016/j.nbd.2012.05.016. 22677032 PMC3392473

[50] Graham RT, Parrish RR, Allberio L, Johnson EL, Owens LJ, Trevelyan AJ. Synergistic positive feedback underlying seizure initiation (Preprint). *bioRxiv* 2021. doi:10.1101/2021.02.28.433224.

[51] Puigserver P, Wu Z, Park CW, Graves R, Wright M, Spiegelman BM. A cold-inducible coactivator of nuclear receptors linked to adaptive thermogenesis. Cell 92: 829–839, 1998. doi:10.1016/S0092-8674(00)81410-5. 9529258

[52] Jiang Z, Rompala GR, Zhang S, Cowell RM, Nakazawa K. Social isolation exacerbates schizophrenia-like phenotypes via oxidative stress in cortical interneurons. Biol Psychiatry 73: 1024–1034, 2013. doi:10.1016/j.biopsych.2012.12.004. 23348010 PMC3638045

[53] Zhou X, Chen Q, Huang H, Zhang J, Wang J, Chen Y, Peng Y, Zhang H, Zeng J, Feng Z, Xu Z. Inhibition of p38 MAPK regulates epileptic severity by decreasing expression levels of A1R and ENT1. Mol Med Rep 22: 5348–5357, 2020. doi:10.3892/mmr.2020.11614. 33174009 PMC7647013

[54] Hu JH, Malloy C, Hoffman DA. P38 regulates Kainic acid-induced seizure and neuronal firing via Kv4.2 phosphorylation. Int J Mol Sci 21: 5921, 2020. doi:10.3390/ijms21165921. 32824677 PMC7460594

[55] Bartley AF, Lucas EK, Brady LJ, Li Q, Hablitz JJ, Cowell RM, Dobrunz LE. Interneuron transcriptional dysregulation causes frequency-dependent alterations in the balance of inhibition and excitation in hippocampus. J Neurosci 35: 15276–15290, 2015. doi:10.1523/JNEUROSCI.1834-15.2015. 26586816 PMC4649003

[56] Chang YT, Chen PC, Tsai IJ, Sung FC, Chin ZN, Kuo HT, Tsai CH, Chou IC. Bidirectional relation between schizophrenia and epilepsy: a population-based retrospective cohort study. Epilepsia 52: 2036–2042, 2011. doi:10.1111/j.1528-1167.2011.03268.x. 21929680

